# Traumatic vertebral artery dissection in an adult with brachial plexus injury and cervical spinal fractures

**DOI:** 10.1186/1749-7221-2-17

**Published:** 2007-09-06

**Authors:** Silas NS Motsitsi, Rian R Steyn

**Affiliations:** 1 Department of Orthopaedic Surgery, Kalafong Hospital, University of Pretoria, Pretoria, South Africa

## Abstract

We present a case of a 32 year-old right-hand dominant woman who sustained a right brachial plexus injury, ipsilateral fractures of the cervical spine transverse processes, and vertebral artery dissection. She presented to us four days following the initiating accident. Magnetic Resonance Imaging showed normal brachial plexus along with vertebral artery dissection with intramural thrombus and vascular lumen occlusion. The dissection was managed conservatively. A repeat CAT-SCAN Angiography three months later showed healing of the dissection plus vascular lumen re-canalization. There were no sequelae due to the dissection.

The details of the case are discussed in this report.

## Background

Cervicocerebral dissection is responsible for strokes in young patients. It accounts for 20% of cerebro-vascular accidents in patients younger than 45 years [1]. Extra-cranial carotid artery dissection accounts for 70%–80% and extra-cranial vertebral artery dissection for 15% of strokes in these young patients. The causes are not completely understood. Triggers of cervico-cerebral dissection are, nose blowing, coughing, chiropractic maneuvers, sudden neck turning, and trauma (minor and major). Genetic (Ehler-Danlos syndrome) and environmental (smoking, hypertension, oral contraceptives and migraine) factors may also be responsible.

Traumatic vertebral artery injury may be occlusive (thrombosis) or non-occlusive (dissection) [2]. The incidence of vertebral artery injury among patients with blunt neck trauma is estimated at 0.20%–0.77% [3]. Major mechanisms of injury are, distraction/extension, distraction/flexion, and lateral flexion. The vertebral artery is easily injured by traction [4]. Only about 12%–24% of unilateral vertebral artery injuries present with signs and symptoms of vertebro-basilar ischaemia. The majority of these injuries are missed because clinicians do not think about them.

Traumatic vertebral artery dissection is common with major penetrating or blunt neck trauma [5]. A case of vertebral artery dissection (VAD) plus brachial plexus injury has been reported in a child following a car accident [6]. There has not been such a case reported in an adult. We report a case of brachial plexus injury, VAD, and ipsilateral five contiguous transverse process fractures of the cervical spine in an adult.

We detail the presentation, physical examination, diagnostic work-up, treatment and follow-up.

## Case presentation

A 32 year-old right hand dominant secretary was involved in a car accident. She was a passenger. The car in which she was traveling was hit from the side by a truck. Her head was thrown into acute left lateral flexion during impact. She immediately felt pain in the neck and partial loss of function of her right upper limb. There was no loss of consciousness. She did not sustain any other injuries. Previous medical history was unremarkable.

She was referred to our Spinal Clinic four days after the accident. She was complaining of painful neck, especially on the right side, and inability to use the right upper limb.

On physical examination, she had torticollis and the affected limb was supported in a sling. The neck was tender from C1-T1 especially on the right side in the posterior triangle. Neurological examination of the right upper limb showed decreased function of the brachial plexus; motor function; C5 = 0/5, C6 = 2/5, C7 = 2/5, C8 = 3/5, and T1 = 4/5 according to the modified MRC scale. There was decreased sensation involving the whole of the brachial plexus distribution. Reflexes were not recorded. The circulation to the limb was normal compared to the opposite side. There were not any other significant findings.

Plain radiographs of the neck (Antero-posterior, lateral, and open-mouth) showed loss of cervical lordosis, fracture of the right transverse process of C6 and increased pre-vertebral soft tissue shadow from C3- C7. Flexion- extension views were done two weeks later (when she was pain-free) and did not show any instability. A computerized tomography scan (CT-SCAN) was requested to exclude other cervical spine fractures. It showed contiguous communited fractures of the cervical transverse processes of C3-C7 on the right side. There were not any other fractures detected.

**Figure 1:**
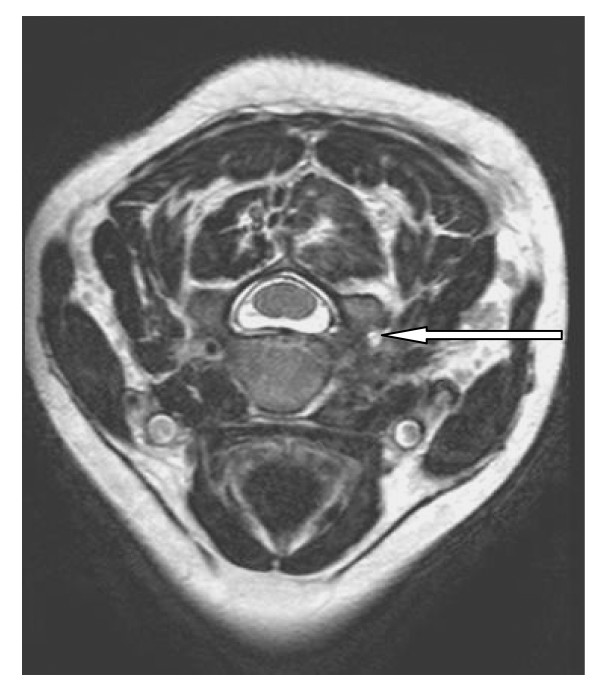
MRI of the cervical spine. T2-weighted image shows high signal intensity (white arrow) of the right vertebral artery. There is an intramural thrombus plus occlusion of the lumen (Grade four dissection). There is no intraluminal thrombosis.

Magnetic Resonance Imaging (MRI) was done to evaluate brachial plexus injury and to exclude vertebral artery injury. [It is our policy to exclude vertebral artery injury in all cases of lateral mass or transverse process fractures of the cervical spine]. The brachial plexus was normal. MRI demonstrated high-signal intensity in both T1- and T2- weighted images of the vertebral artery on the right side. There was intramural methaemoglobin plus occlusion of the lumen, but there was no intraluminal thrombus. There was no intimal flap demonstrated (Figure 1). This was in keeping with vertebral artery dissection. The spinal cord was normal.

On the advice of the physicians, she was placed on prophylactic treatment: Aspirin 650 mg orally twice a day for three months. We were advised to repeat angiography in three months. She was referred to the brachial plexus clinic for follow-up.

**Figure 2:**
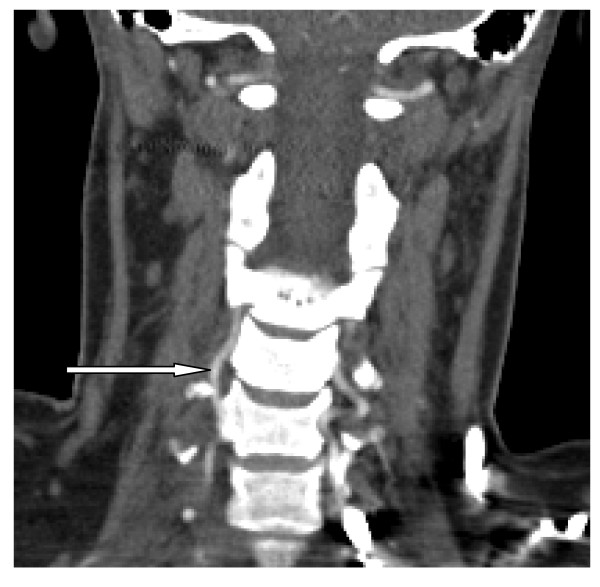
CTA done three months later using a 64-slice coronal reconstruction. There is normal blood flow at the level of C4 to C2 (white arrow).

We repeated angiography three months later. She was evaluated using a 16-channel multi-detector CT SCAN. The scan showed complete healing of the dissection and recanalization of the right vertebral artery (Figure 2). She continued her further management at the brachial plexus clinic. They explored the brachial plexus surgically but did not find any neuromas or pathology needing reconstruction. They made a decision to manage her conservatively.

## Discussion

Our patient presented with a devastating injury involving her dominant limb. She was referred because of neck pain and brachial plexus palsy. The brachial plexus injury dominated the clinical picture. There was a potentially devastating injury which was not suspected: vertebral artery dissection. This injury is commonly overlooked. The clue to this injury was a transverse process fracture of C6 which was not diagnosed in the original X-rays. The full extent of the injuries was only picked up during re-evaluation at our clinic. The most likely mechanism of fractures of the transverse processes was avulsion or traction which occurred during forceful lateral neck flexion. Prophylactic treatment with Aspirin was pre-emptive. She made a good recovery of the vertebral artery dissection: the artery re-canalized and the dissection healed. There were no neurological sequelae attributable to VAD.

The areas of the vertebral artery vulnerable to injury during blunt neck trauma are, V2 (inside the transverse foramina) and the V3 (between the C1 and the base of the skull) [7]. The latter is usually injured in minor trauma. Clay Cothren et al. [8] in a large series concluded that the following cervical spine injury patterns mandate screening for blunt cerebro-vascular injury; fractures extending into the transverse processes, subluxations, and fractures of the upper cervical spine. Other authors have argued that fractures and fracture-dislocations also warrant exclusion of injuries of the vertebral artery. According to Hiroshi Taneishi et al. [9] VAD occurs in 20% of patients with cervical spine fractures or fracture-dislocation. They found that all their patients who had VAD had spinal cord injury: there was no significant correlation between the two. However, there was a statistically significant correlation between unilateral facet dislocation and vertebral artery occlusion. They also noted that occlusion secondary to VAD can recanalize in up to 85% of cases within three months by spontaneous mechanisms. Philip J. Torina et al. [10] in their series found that vertebral artery occlusion is significantly more common in motor-complete spinal cord injury.

One of the most controversial issues in traumatic cerebro-vascular trauma is what is the best modality for investigating blunt cerebro-vascular injury. The gold standard is Digital Subtraction Angiography (DSA). The problem with DSA is that it is an invasive procedure. Other modalities available are MRI, MRI-Angiography and multi-detector CT-Angiography (CTA). Lawrence D. Bub et al. [11] in his series of 32 patients concluded that the accuracy of CTA in vertebral injury remains in question. It was Alexander L. Eastman et al. [12] in a large series of 162 patient who demonstrated that CTA is a very good screening tool for blunt cervical injury. They demonstrated that the overall sensitivity, specificity, positive predictive value, negative predictive value, and accuracy of CTA for the diagnosis of blunt cerebro-vascular injury were 97.7%, 100%, 100%, 99.3%, and 99.37, respectively.

The natural history of VAD is unknown. It can heal spontaneously, it can develop occlusion or it can form a pseudo-aneurysm. The clinical significance of VAD lies in its potential to form intra-luminal thrombosis and this has potential for embolization. Vertebral artery injury (thrombosis or dissection) can lead to basilar stroke which has a poor prognosis. The mortality rate due to vertebro-basilar ischaemia can reach 75%–86% [8]. Treatment for VAD is controversial; it not clear whether patients must be heparinized, be treated with antiplatelets (Aspirin) or treated at all. Izhar Hasan et al. [13] in their review of 68 children found that the most common treatment for VAD was antiplatelet therapy. They found that asymptomatic recovery occurred in 12 of 15 (80%) children who received antiplatelets therapy compared to 4 of 15 (27%) who received anticoagulation therapy with or without antiplatelet. Once thrombosis occurs, it is also controversial whether anticoagulation or antiplatelet therapy should be the treatment of choice. Vadim Beletsky et al [14] showed that the recurrence rate for embolization is decreased significantly (by 8.3%) in patients on anticoagulation compared to those on Aspirin (12.4%). This difference in outcome at one year was not statistically significant. It is prudent to seriously consider prophylactic treatment (unless contra-indications exist) because the prognosis for brainstem ischaemia is very poor.

## Conclusion

Based on the literature and on this case report, we make the following recommendations;

■ Vertebral artery injury must be excluded in high-risk cases.

■ Prophylactic treatment for VAD must be seriously considered unless there are contra-indications

A randomized control trial is required (if ethically acceptable) comparing prophylactic treatment versus non-treatment in patients with VAD.
